# Supplementary material for: Direct observations of airflow separation over ocean surface waves
Source: Nat Commun. 2025 Jul 1;16:5526. doi: 10.1038/s41467-025-61133-1 (PMC12214478; doi:10.1038/s41467-025-61133-1)
Supplement: Supplementary file 1 — Supplementary Information [file 41467_2025_61133_MOESM1_ESM.pdf]

Supplementary Material for

Direct observations of airflow separation over ocean surface waves

Marc P. Buckley, Jochen Horstmann, Ivan Savelyev, Jeff R. Carpenter

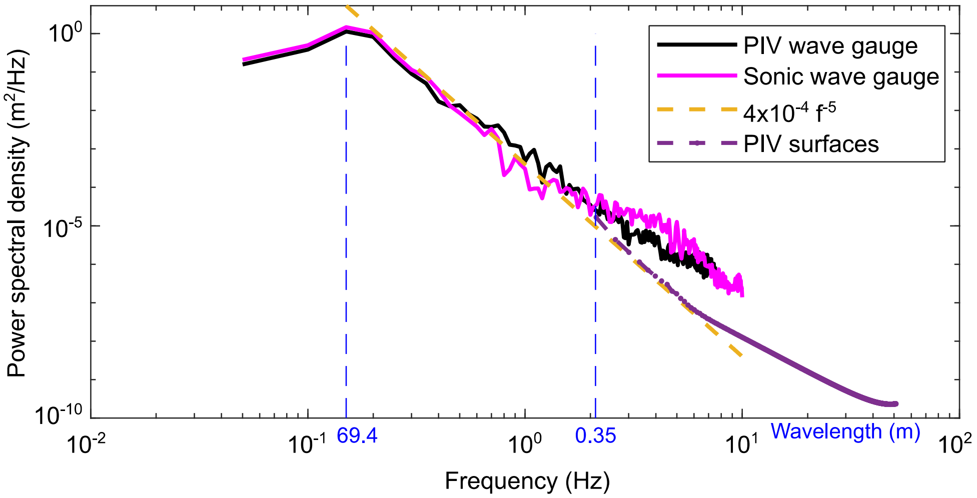

Fig. S1: Frequency spectra of the water surface elevation from different data sources. The PIV surfaces provide 2 frequency spectra, the first from a time series of the middle of the surface detected on each PIV image (black, noted "PIV wave gauge"), the second from the wavenumber spectrum of each spatial surface snapshot, converted to frequency space using the dispersion relation (purple, noted "PIV surfaces"). The magenta line shows the frequency spectrum obtained from the single point time series of water surface elevation measured by the sonic wave gauge. The dashed orange line is proportional to f^{-5} (where f is the frequency).

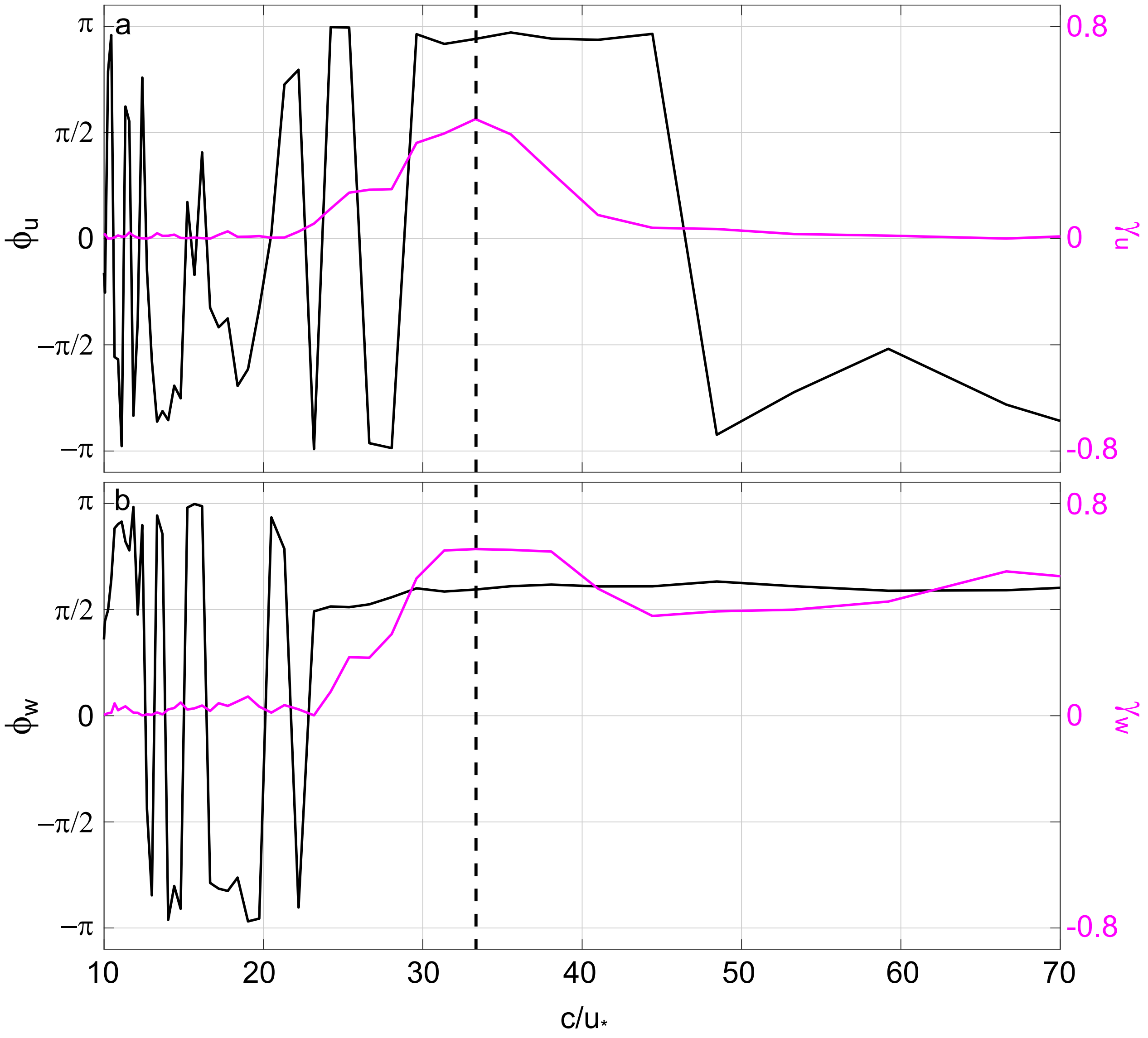

Fig. S2: Cross-spectral analysis of wave gauge and near-surface anemometer measurements. Represented are the phase shift (ϕ, black solid line) and normalized coherence function (γ, magenta) of the cross-spectral density between time series of the water surface elevation and anemometer velocities (at 2.6 m above the mean water level), as a function of wave age. The vertical dashed line represents the peak correlation value (which matches the peak wave age). Both the horizontal (panel a) and vertical (panel b) components of the airflow velocities are considered.

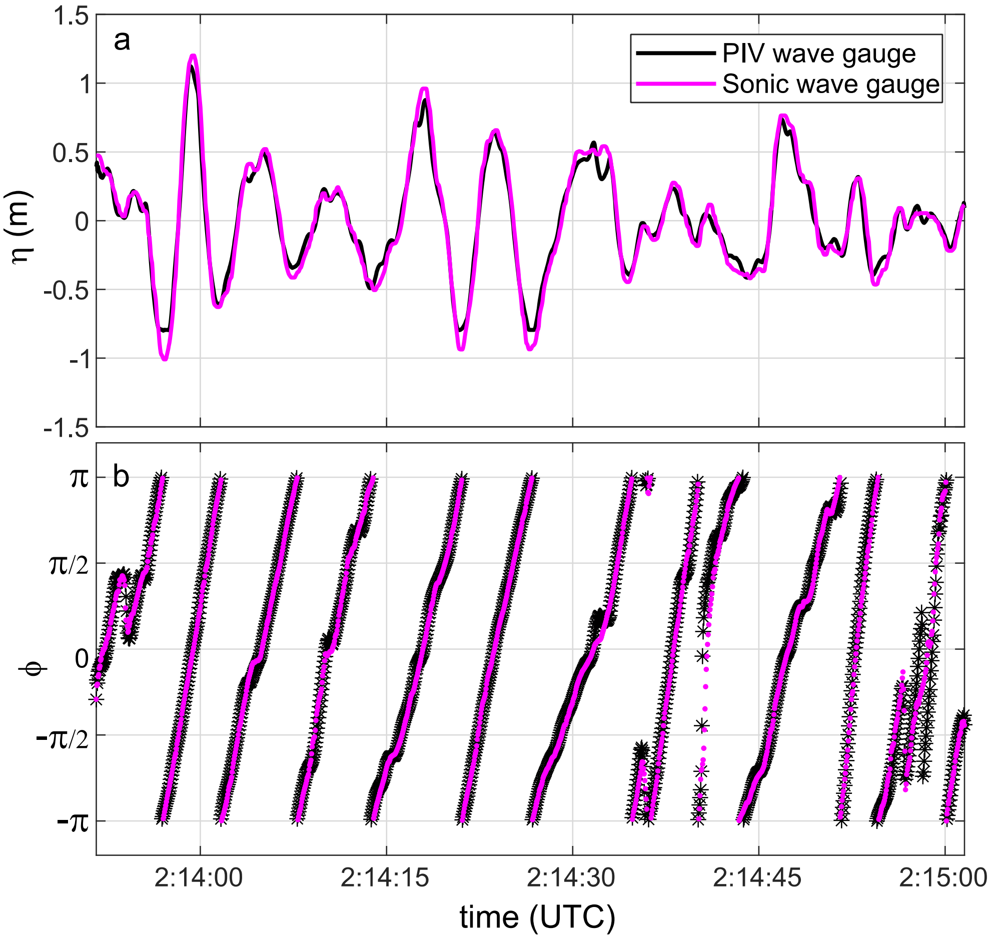

Fig. S3: Wave phase detection for the dominant (fast) waves. a: Water surface elevations from PIV images (black) and from the sonic wave gauge (magenta). b: Corresponding instantaneous wave phases (ϕ), obtained from a Hilbert transform of the water surface elevation time series.

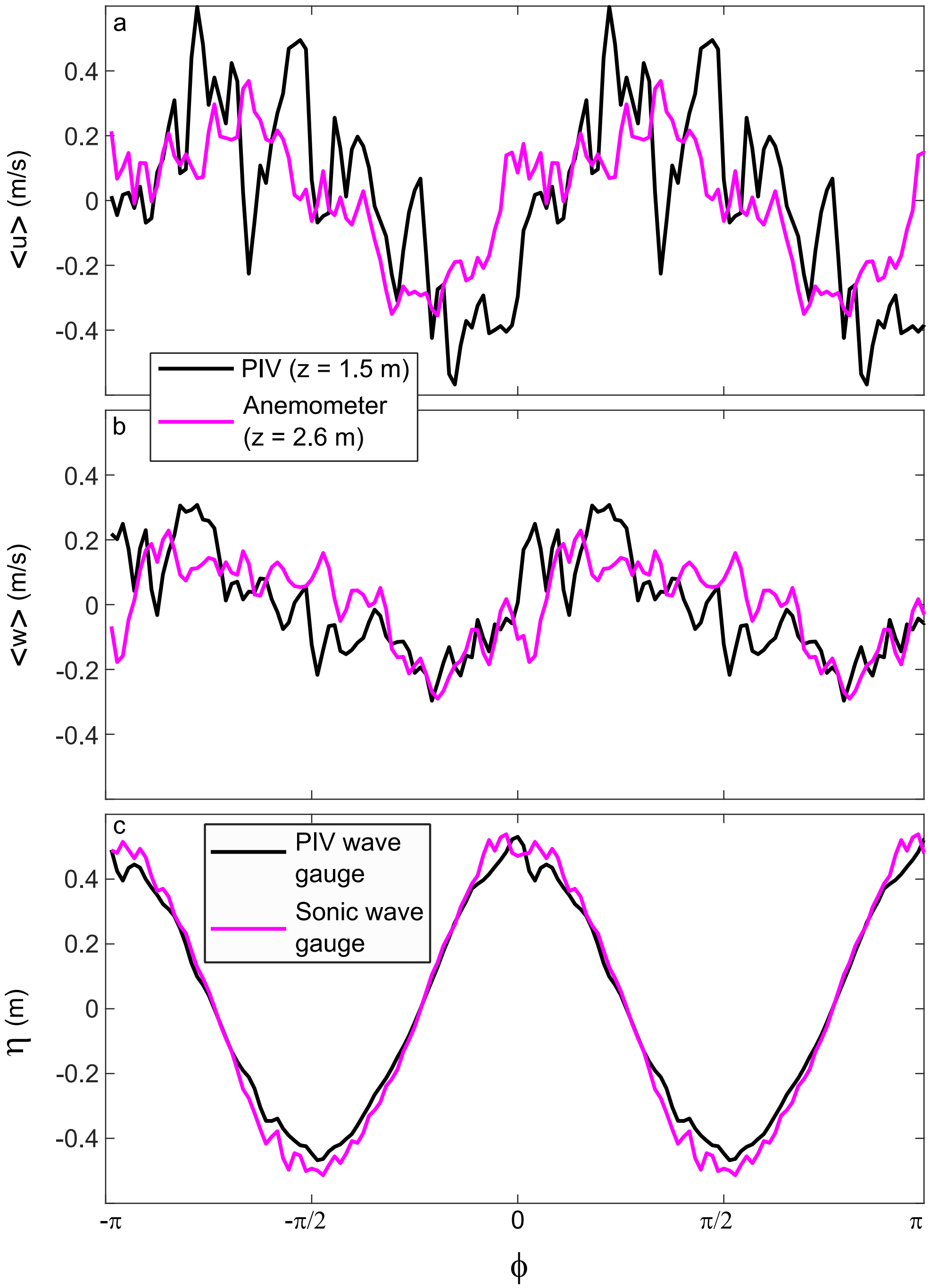

Fig. S4: Comparison of phase averaged quantities from PIV imagery vs in situ sensors. a (resp. b): Phase averaged horizontal (resp. vertical) velocities from PIV (black) and sonic anemometer (magenta) measurements. c: Phase averaged water surface elevations, obtained from raw PIV images (black) and from sonic wave gauge (magenta) measurements.
